# Advances in Carbon-Based Aerogels for CO_2_ Capture: Fundamental Design Strategies and Technological Progress

**DOI:** 10.3390/gels11050361

**Published:** 2025-05-14

**Authors:** Shakila Parveen Asrafali, Thirukumaran Periyasamy, Gazi A. K. M. Rafiqul Bari

**Affiliations:** 1 Department of Fiber System Engineering, Yeungnam University, 280 Daehak-Ro, Gyeongsan 38541, Gyeongbuk, Republic of Korea; 2 Department of Mechanical Engineering, Gachon University, 1342 Seongnam-daero, Sujeong-gu, Seongnam-si 13120, Gyeonggi-do, Republic of Korea

**Keywords:** aerogel, carbon, microstructure, porous carbon, CO_2_ capture

## Abstract

Carbon-based aerogels have garnered significant attention for CO_2_ capture owing to their low-cost precursors, tunable structures, and high porosity. Their performance in CO_2_ adsorption is intricately linked to their microstructural and textural features, including pore size distribution, surface area, and surface chemistry. Micropores (<2 nm) are particularly effective due to their size compatibility with CO_2_ molecules, while surface functional groups enhance adsorption through hydrogen bonding and electrostatic interactions. Strategic design approaches have focused on tailoring these properties to optimize CO_2_ uptake under realistic conditions. This review provides a comprehensive overview of recent advancements in the structural engineering of carbon aerogels, emphasizing the role of hierarchical porosity and heteroatom doping (nitrogen, oxygen, sulfur, etc.) in enhancing adsorption capacity and selectivity. Experimental and theoretical studies have highlighted how the synergistic control of microstructure and surface chemistry leads to superior adsorption performance. Furthermore, this review identifies current challenges, such as limited structural stability and insufficient mechanistic understanding, which hinder further progress. Future research directions are proposed, including advanced pore architecture control, functional group engineering, and the integration of in situ characterization techniques. Overall, this review serves as a guide for the rational design of next-generation carbon-based aerogels tailored for efficient and scalable CO_2_ capture technologies.

## 1. Introduction

The continuous emission of CO_2_ worldwide has significant environmental impacts that threaten human survival, necessitating global regulatory strategies. Although cumulative efforts focus on reducing CO_2_ emissions, ongoing industrialization, development, and reliance on fossil fuels globally pose challenges to achieving effective CO_2_ control. Atmospheric CO_2_ levels have already reached approximately 420 (280 ppm, part per million from pre-industrial levels) ppm, and projections indicate they could rise to 550 ppm by 2050 (32% increase compared to present) [1]. Given these trends, a net-zero strategy is crucial for creating a sustainable environment. This strategy aims to balance CO_2_ emissions by removing an amount equivalent to what is released, thereby meeting human energy demands while mitigating environmental impacts. Achieving this goal requires strategic CO_2_ capture methods, whether through point-source capture at industrial sites (power plant, cement industry, steel industry) or direct air capture (DAC) [2,3]. It is estimated that planting half a trillion trees would only sequester a fraction of the necessary CO_2_—around 205 gigatons—underscoring the need for an efficient, multifaceted approach (carbon pricing, renewable energy, energy efficiency, carbon capture and storage, sustainable agriculture, geoengineering) with clearly defined climate goals [4,5,6,7,8,9,10].

Various research efforts have focused on advancing CO_2_ capture technology, with liquid amine capture, a century-old state-of-the-art method, achieving high maturity levels. This technology demonstrates a substantial working capacity of ~1.5 mmol g^−1^, a rapid absorption rate (0.05–0.18 mol m^−2^ s^−1^), a capture rate of 90% for 30 wt.% monoethanolamine (MEA), and suitability across a wide concentration range (4–5 vol.% CO_2_) [2,11]. Despite these advantages, liquid amine systems encounter significant challenges in industrial applications worldwide. Thermal degradation at desorption temperatures exceeding 100 °C and oxidative degradation from reactive contaminants such as O_2_, SO_x_, and NO_x_ contribute to operational inefficiencies. Additionally, severe corrosion affects steel reactor vessels and pipes, and solvent degradation can produce harmful byproducts, including carcinogenic nitrosamines and nitramines with substantial solvent losses (2.6 kg per ton of CO_2_ capture) [12,13,14,15]. Furthermore, the regeneration process demands a high energy input of approximately 3–5 GJ per ton of captured CO_2_ (60–80% of the total energy consumed during CO_2_ capture process) [13,16,17]. These drawbacks have led to a global search for alternative, sustainable CO_2_ capture methods, such as solid adsorbents, to overcome these limitations effectively.

Solid adsorbents present substantial potential to address the limitations of liquid amine systems, offering superior thermal and mechanical stability and displaying promising working capacities (1.5–6 mmol g^−1^). Additionally, they have lower heats of adsorption compared to liquid amine solvents, which exhibit an adsorption enthalpy of approximately 84 kJ mol^−1^, enabling efficient operation through either pressure swing or temperature swing adsorption. Notably, materials such as metal–organic frameworks (MOFs), zeolites, covalent–organic frameworks (COFs), and carbon-based adsorbents display heats of adsorption around 50, 30–50, and 20–30 kJ mol^−1^, respectively [18,19,20,21]. Among solid adsorbents, carbon-based materials are particularly promising due to their tunable porous structures, cost-effectiveness, chemical and mechanical stability, and adaptability to a broad pressure range—from high pressures exceeding 35 bar to low pressures below 1 bar. This includes conditions for direct air capture (DAC) following amine modification. Furthermore, these materials can be engineered for selective adsorption over competing gases, are highly reusable without performance degradation, and require less energy for regeneration due to balanced isosteric heats of adsorption (20–30 kJ mol^−1^) [22,23,24,25].

Carbon-based aerogels are emerging as promising materials for carbon capture due to their three-dimensional, multiporous architecture, high surface area, low density, and tunable properties—elements which significantly impact CO_2_ capture efficiency. The chemical and thermal stability of carbon aerogels enhances their durability, allowing for long-term performance in high-temperature applications. Furthermore, these aerogels can be synthesized from low-cost raw materials, including biomass and waste products, making them cost-effective for large-scale manufacturing [26,27,28]. Fine-tuning the microstructure and textural properties of carbon aerogels is achievable by adjusting synthesis parameters such as raw material selection, solvent choice, dissolution and gelation conditions, drying methods, carbonization processes, and activation techniques involving acids, bases, salts, or gases. Achieving an optimized hierarchical pore structure that balances ultra-, micro-, meso-, and macro-porosity is critical for maximizing CO_2_ capture while ensuring rapid adsorption kinetics [29,30].

The effective surface modification of carbon aerogels is also essential for selective gas adsorption in complex gas mixtures. However, improper surface treatments or pore designs can impede surface diffusion and intra- or inter-pore diffusion, thereby limiting capture efficiency [2,29]. Despite these advantages, carbon aerogel manufacturing remains complex and involves multi-step processes that are challenging to scale for practical applications. This review provides an in-depth analysis of the key criteria for efficient CO_2_ adsorption, including pore structure optimization, surface functionalization, and material stability under diverse conditions. Additionally, it outlines future research directions, such as developing novel carbon materials with customized microstructures and leveraging advanced characterization techniques to enhance our understanding of adsorption mechanisms. Collectively, this review aims to guide researchers in designing and synthesizing next-generation carbon-based materials with enhanced CO_2_ capture capabilities.

## 2. Fundamentals of Carbon Aerogels

Aerogels, often referred to as “frozen smoke”, “solid air”, or “blue smoke”, are lightweight materials characterized by a three-dimensional scaffold permeated with air. These low-density solids are created by replacing the liquid component in a gel with gas, resulting in a highly porous structure containing ultra-, micro-, meso-, and macropores. Typically, the preparation of aerogels involves several precise steps, beginning with sol preparation, which uses metal oxide or polymer precursors to form a colloidal suspension in a solvent. This sol undergoes polymerization to create a solid network filled with solvent, a stage known as gelation. Gel formation occurs via processes like polycondensation and polyesterification, which increase the viscosity of the solution [31,32].

To improve structural integrity, the gel undergoes aging, which stabilizes the structure and prevents cracking. The original solvent is then exchanged for a more volatile solvent to facilitate the drying process. Drying is a critical step that is needed to preserve the desired shape and can involve supercritical drying, freeze-drying, or thermal drying. To achieve specific properties, the aerogel is subjected to carbonization—a thermal treatment performed at high temperatures—followed by activation. Activation can be carried out using various techniques, including acid, base, or salt treatments, and templating with soft, hard, or gas templates. These processes produce carbon aerogels with tailored properties, making them versatile materials for applications requiring high surface area, structural stability, and tunable porosity [32,33].

**Figure 1:**
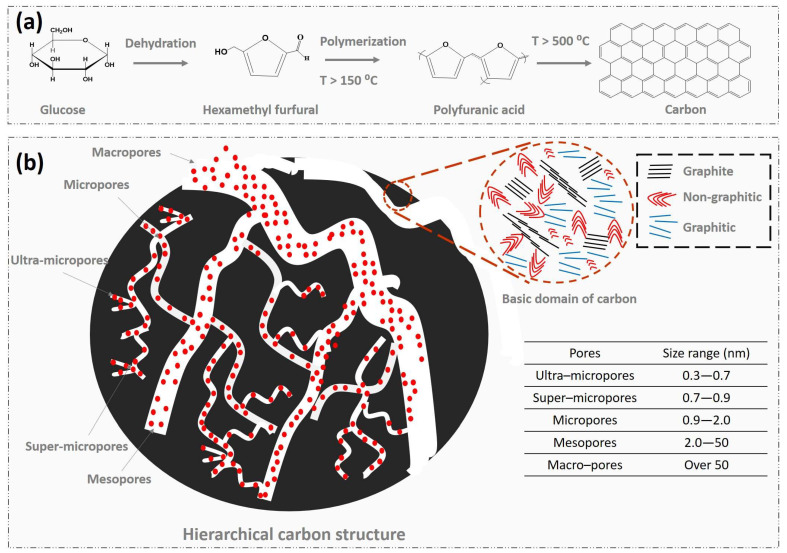
(**a**) Schematic representation of carbonization of organic components, using glucose as reference, and (**b**) illustration of hierarchical porous carbon, showing primary pore size (ultra- to macro-sized pores) with basic domains forms.

The carbonization of organic precursors at high temperatures (400–1100 °C) produces carbon structures featuring multi-phase graphitization, encompassing graphite, non-graphitic, and graphitic domains in both amorphous and crystalline forms (Figure 1). The crystalline phase is categorized into turbostratic (T–C) and orthogonal graphitic (G–C) structures, with an approximate interlayer spacing of 0.34 nm. The carbonization process involves stages such as dehydration, intramolecular condensation, and decarboxylation, yielding various intermediate products [2,34,35]. At higher temperatures, aromatic polycondensation occurs, generating both amorphous and crystalline phases. The choice of precursor, the intermediate products generated, and variations in carbonization conditions (heating rate, gas flow, carbonization area, precursors amount, etc.) significantly influence the resulting textural, structural, and microstructural properties [2,36,37]. During polycondensation, gases like ammonia, methane, carbon monoxide, carbon dioxide, hydrogen, and water vapor are released. These act as gas templates, creating internal pores and influencing the crystalline and amorphous phases, the degree of aromatic condensation, lateral size (La), crystallite size (Lc), stacking number, and interlayer spacing. Further structural tuning can be achieved through various activation methods: (i) physical activation using gases (e.g., CO_2_, NH_3_, steam, He, Ar, air, or binary mixtures), (ii) chemical activation with compounds like HCl, FeCl_3_, ZnCl_2_, H_3_PO_4_, H_2_SO_4_, HNO_3_, KOH, NaOH, CaCO_3_, K_2_CO_3_, H_2_O_2_, and KMnO_4_, (iii) metal ion activation (e.g., Li^+^, K^+^, Na^+^, Rb^+^, Cs^+^), (iv) the use of hard templates such as zeolites, porous metals, metal powders, metal foams, silica, and MOFs, (v) the use of salt templates (e.g., molten salts like NaCl, ZnCl_2_, and eutectic salts), and (vi) the use of soft templates (e.g., Pluronic F108, Pluronic F127, P123, and ZIF–8, molecular assembled organic compounds) [11,38,39,40]. These methods enable precise control over the formation of multi-scale pores from ultra- to macro-sized, directly enhancing CO_2_ capture performance and kinetics by creating structures selectively tailored to accommodate the 0.33 nm kinetic diameter of CO_2_ [35].

## 3. Carbon Aerogels Design Strategies

**Figure 2:**
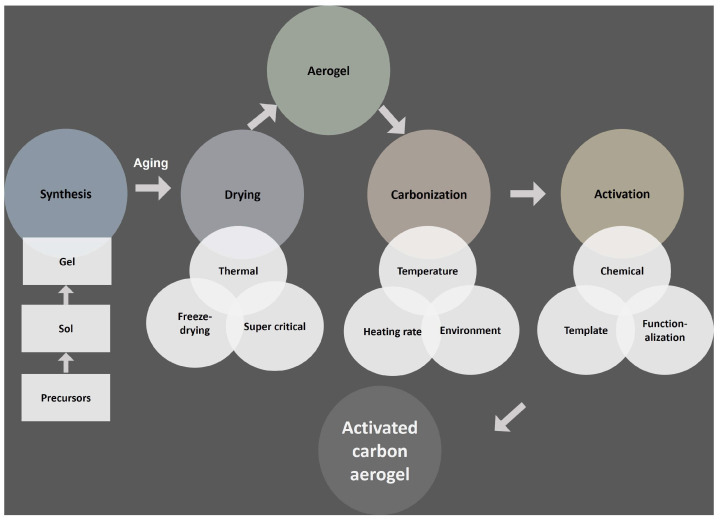
Schematic of fabrication of activated carbon aerogel.

Designing carbon aerogels for effective CO_2_ capture requires a precise and technical approach to achieve specific, desirable properties. Key factors include synthesis parameters, drying techniques, carbonization, and activation processes; each of these plays a crucial role in tailoring the material for optimal performance (Figure 2). During synthesis, parameters such as precursor type, concentration, precursor ratio, pH, solvent type, and temperature must be carefully controlled to influence hydrolysis rates, nanoparticle formation, and gel structure [6,41,42]. The choice of drying method—whether supercritical, freeze-drying, or thermal drying—affects the aerogel’s structural integrity and mechanical stability. In the carbonization stage, conditions such as temperature, atmosphere, gas flow rate, heating rate, and duration impact pore size and distribution across ultra-, micro-, meso-, and macro-sized pores [42,43]. Activation processes further refine pore structure and include chemical activation (with acids or bases) as well as templating techniques (salt, soft, or hard templates) [43,44]. Surface functionalization through heteroatom doping (with elements like S, N, P, O, and B) can enhance selectivity in mixed-gas environments. The following sections present a detailed discussion on recent research strategies for and multi-faceted approaches to optimizing carbon aerogels in CO_2_ capture applications [2].

### 3.1. Carbon Aerogels Form Biomass

**Figure 3:**
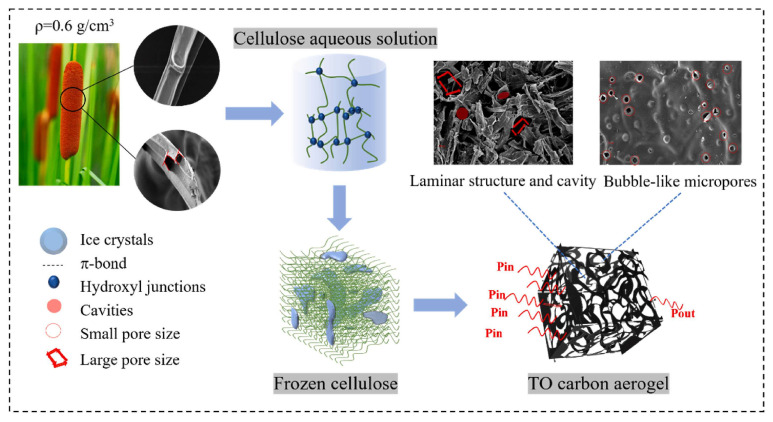
Schematic of fabrication of porous cellulose carbon aerogels from Typa Orientalis biomass. Reproduced with permission. Copyright 2022, Elsevier Ltd. [47].

Biomass-based sources are increasingly recognized as promising resources for producing carbon aerogels due to their low cost and abundant availability. Cellulose, a biopolymer present in biomass, serves as an excellent precursor for carbon aerogel synthesis due to its renewability, biocompatibility, low density, high porosity, and large surface area [45,46]. Cheng and colleagues developed high-value-added, ultra-lightweight carbon aerogels from Typa Orientalis (TO) [47]. The TO cellulose was extracted from TO fibers via acid hydrolysis, preserving the original network structure (Figure 3). This process isolated cellulose (40–50%) by separating it from hemicellulose (a polysaccharide, 20–30%) and lignin (20–30%), which act as binders for cellulose fibers [45]. The TO cellulose was then freeze-dried (−53 °C, 0.1 MPa) to form cellulose aerogels, followed by carbonization at 780 °C to obtain carbon aerogels. The results show that activation process significantly improves the adsorption properties of carbon aerogels, resulting in a higher specific surface area of 1840 cm^2^ g^−1^ with graded microporous structure and more abundant surface functional groups. Pore sizes of approximately 2 and 3.5 nm were introduced by activating the carbon aerogels with NaOH in a 3:1 mass ratio. The resulting carbon aerogels demonstrated a CO_2_ capture capacity of ~15 mmol g^−1^ at 30 bar (25 °C), along with a hydrogen storage capacity of 0.6 wt.% at room temperature and the adsorption of volatile organic compounds such as o-xylene (123.31 mg/g) and o-dichlorobenzene (124.57 mg/g) [47].

**Figure 4:**
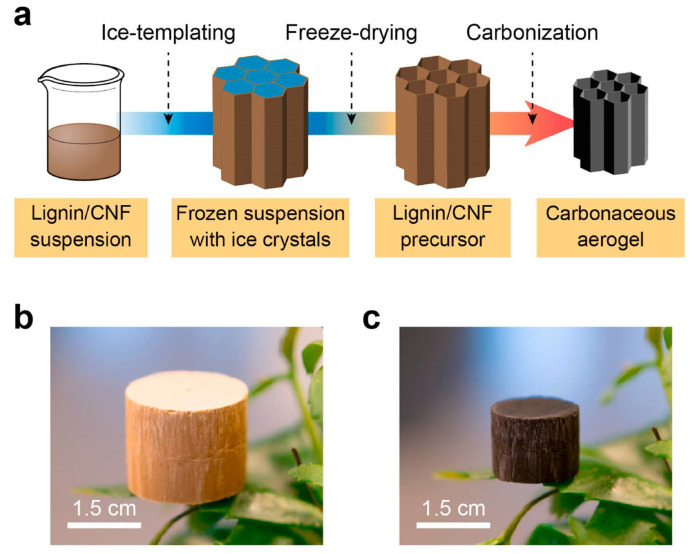
(**a**) Schematic preparation of carbon aerogels, (**b**) lignin/CNF precursor, and (**c**) carbon aerogel. Reproduced with permission. Copyright 2021, Elsevier Inc. [48].

In another approach, Geng and colleagues used lignin as a primary component with cellulose nanofibers (CNFs) to develop monolithic carbon aerogels, combining ice templating and carbonization. Different reaction parameters, including CNF concentration (lignin: CNF = 85: 15), solid content (3 wt.%) and carbonization time (1 h), produced suitable carbon aerogel structures with less wrinkled morphologies, smoother cell walls, porous structures (pore size of 2.8 nm), and enhanced surface areas (400 m^2^ g^−1^) (Figure 4) [48]. These monolithic carbon aerogels exhibit a homogeneous, porous, anisotropic hierarchical structure with tracheid-like macropores aligned in a single direction, facilitating rapid CO_2_ sorption kinetics [49]. The anisotropic structure, flexible in both axial and radial directions, enhances the aerogel’s performance as an adsorbent for CO_2_. This configuration is especially advantageous for use in packed columns for CO_2_ capture, as it does not require binders, offering a promising pathway for scalable and widespread application. The monolithic carbon aerogels achieved a CO_2_ capture capacity of 4.5 mmol g^−1^ at 25 °C and 1 bar [48,50].

The Zhong and Peng group developed hierarchical porous carbon aerogels with desirable macropores, mesopores, and micropores from cellulose by utilizing a dissolution and gelation process in specific solvents [51]. Dissolution and gelation disrupt the compact structure of cellulose fibers, enabling their reconstruction into a tunable porous architecture based on the solvent properties, thereby directing pore size distribution. Cellulose dissolves in the solvent (NaOH, LiOH, and urea) and forms a gel through aggregation driven by hydrogen bonding among cellulose chains in an anti-solvent environment (H_2_SO_4_, acetone, methanol, ethanol, and butanol) [52,53]. This approach allows for the development of multidimensional intermediate structures, which can be further carbonized to produce carbon aerogels. The activation of carbon aerogel in a CO_2_ atmosphere (Cell-CO_2_) resulted in enhanced CO_2_ uptake of 3.4 mmol g^−1^ at 25 °C and 1 bar with an enlarged surface area (1892 m^2^ g^−1^) and a desirable micropore volume (0.37 cm^3^ g^−1^) [51].

**Figure 5:**
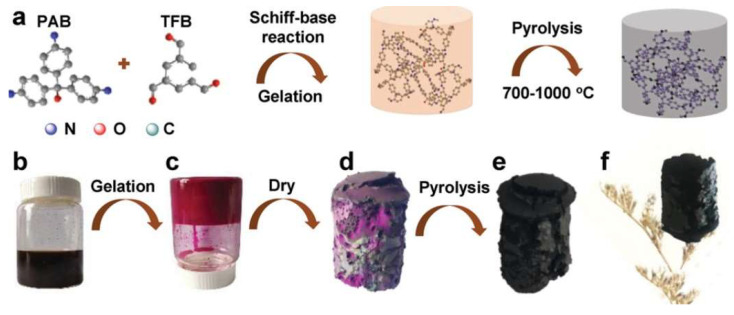
(**a**) Illustrations of NCA synthesis, (**b**) polymer reaction solution, (**c**) polymer gelation, (**d**) polymer aerogel, (**e**) NCA after pyrolysis, and (**f**) NCA with standing-up pistils. Reproduced with permission. Copyright 2019, WILEY-VCH Verlag GmbH & Co. KGaA, Weinheim [54].

The Liao group developed N-doped carbon aerogels (NCAs) by pyrolyzing a porous organic polymer derived from the pararosaniline base and 1,3,5-triformylbenzene via a Schiff-base reaction through hydrothermal gelation (Figure 5) [54]. This preparation method eliminates the need for complex drying techniques such as freeze-drying or supercritical drying. It was found that the pyrolysis temperature of 1000 °C was effective in producing porous NCA (NCA-1000) with a low bulk density of 5 mg cm^−3^, a high surface area of 2356 m^2^ g^−1^, and suitable micropore volume of 0.35 cm^3^ g^−1^, exhibiting a CO_2_ uptake capacity of 6.1 mmol g^−1^ at 0 °C and 1 bar, and one of 33.1 mmol g^−1^ at 50 °C and 30 bar. This study identifies micropores with a size of 0.7–0.8 nm as critical for CO_2_ uptake at low pressures (1 bar) and lower temperatures (0–25 °C). Additionally, the study demonstrates that selectivity (CO_2_/N_2_) decreases as pyrolysis temperature increases. Under typical flue gas conditions (15% CO_2_ by volume balanced with N_2_), carbon aerogels pyrolyzed at 900 °C show selectivity values of 137 and 47 at 0.15 and 1 bar, respectively.

### 3.2. High-Pressure CO_2_ Adsorption for Pre-Combustion Capture Using Aerogels

The sorption-enhanced reaction (SER) process is employed for pre-combustion CO_2_ capture and hydrogen generation, effectively enhancing hydrogen yield by in situ CO_2_ removal during the water gas shift reaction or the steam reforming of methane. The SER process operates at relatively high temperatures (200–500 °C) and pressures (1–30 bar). Conventional adsorbents used in this process, such as zeolites, activated carbon, lithium zirconates, and calcium oxide, exhibit low CO_2_ capacities (0.2–1 mmol g^−1^), poor selectivity, and slow adsorption kinetics, requiring substantial energy for regeneration [9,49,55].

**Figure 6:**
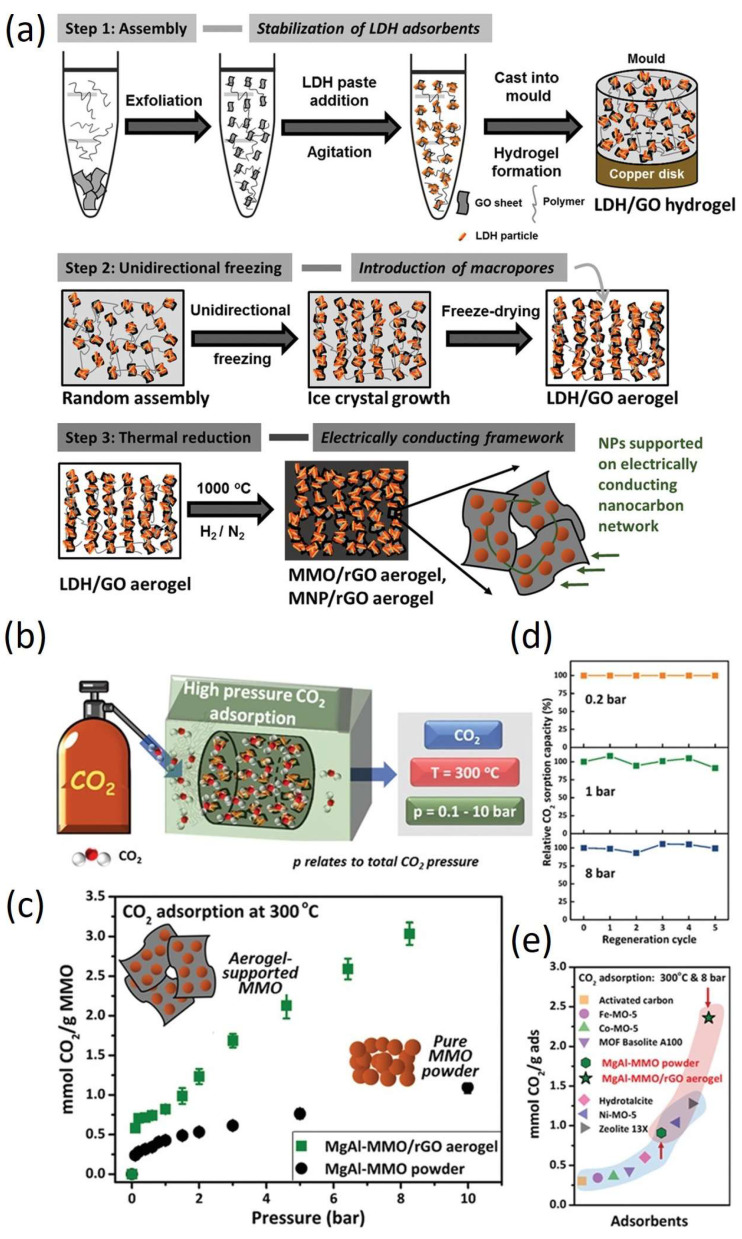
(**a**) Schematic illustration of synthesis process for electrically conductive sorbent–nanoparticle/rGO hybrid aerogel, with MMO representing mixed metal oxide and MNP denoting metal nanoparticles; (**b**) depiction of CO_2_ adsorption under high-pressure conditions on MgAl–MMO/rGO aerogels; (**c**) CO_2_ adsorption isotherms for MgAl–MMO powder and MgAl–MMO/rGO aerogel measured at 300 °C across a pressure range of 0.2–10 bar; (**d**) CO_2_ capacity retention performance of MgAl–MMO/rGO aerogel over five regeneration cycles at 400 °C under CO_2_ pressures of 0.2, 1, and 8 bar; and (**e**) comparison of high-pressure CO_2_ sorption capacities (8 bar, 300 °C) of MgAl–MMO powder and MgAl–MMO/rGO aerogel with those of other sorbent materials. Reproduced with permission. Copyright 2020, WILEY–VCH Verlag GmbH [56].

Layered double hydroxide (LDH)-derived mixed metal oxides (MMOs) have shown some improvement in capacity; however, challenges remain regarding their adsorption kinetics, long-term stability, and loss of capacity due to particle sintering after successive sorption cycles. In response, the Menzel group developed 3D-structured macroscopic reduced graphene oxide (rGO) aerogels with LDH as suitable supports for MMO nanoparticles (Figure 6) (T = 300 °C, p_CO2_ = 1–30 bar) [56]. These rGO aerogels serve as electrically responsive platforms for LDH-derived nanoparticles, creating multifunctional porous materials. The resistive heating of rGO aerogels allows for the faster regeneration of the nanoparticles. Under elevated conditions (300 °C, 8 bar), Mg–Al–MMO nanoparticles demonstrated a CO_2_ adsorption capacity of 2.36 mmol g^−1^, significantly outperforming previous materials (of 0.80 mmol g^−1^). The study clearly evidences that rGO aerogels provide excellent stabilizing support for MgAl-MMO, showing exceptional CO_2_ uptake under elevated conditions [56].

### 3.3. Effects of Pores and Surface Properties

**Figure 7:**
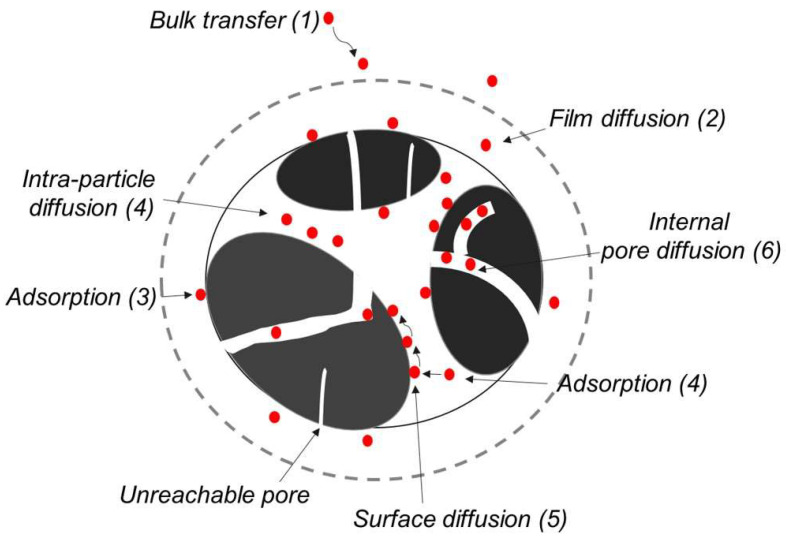
Schematic description of CO_2_ (red) adsorption process on porous carbon (black). Reproduced with permission. Copyright 2023, Springer Nature, Bari et al. Korean Carbon Society [2].

Various studies have focused on optimizing pore size to efficiently accommodate CO_2_ molecules. Additionally, the surface properties of materials can influence the diffusion of CO_2_ due to its high polarizability (26.3 × 10^−25^ cm^3^) and quadrupole moment (13.4 × 10^−40^ C m^2^), which can enhance CO_2_ selectivity over N_2_, which has a lower polarizability (17.6 × 10^−25^ cm^3^) and quadrupole moment (4.7 × 10^−40^ C m^2^) [2]. It is also important to consider the weak acidic nature of CO_2_ and its interaction with functionalized surfaces through acid–base interactions, electrostatic quadrupole interactions, and hydrogen bonding. These factors affect the overall adsorption scenario and the complete molecular structure of the adsorbent (Figure 7). Different functional groups modify the adsorption energy, for example, oxygen-containing groups exhibit adsorption energies of −14.3 to −22.6 kJ mol^−1^, while nitrogen-containing groups show higher energies, ranging from −22.1 to −27.1 kJ mol^−1^, compared to pure carbon (−6.6 kJ mol^−1^) [2]. Among the different nitrogen configurations, pyridinic, pyrrolic, and graphitic nitrogen have distinct effects on adsorption performance. Pyridinic nitrogen, located at the edges of graphene planes, possesses a lone pair of electrons and acts as a strong Lewis base. This configuration facilitates acid–base interactions with the weakly acidic CO_2_ molecules, enhancing adsorption through strong chemisorptive binding. Studies have shown that pyridinic-N contributes significantly to increased isosteric heat of adsorption, often in the range of −25 to −30 kJ mol^−1^, indicating strong binding affinity. Pyrrolic nitrogen, found in five-membered heterocycles, introduces localized electron density and defects that modulate the electronic structure of the carbon matrix. While less basic than pyridinic-N, pyrrolic-N still participates in weak hydrogen bonding and contributes to electrostatic interactions with the CO_2_ quadrupole moment. Its presence can enhance the overall adsorption capacity, particularly under low-pressure conditions, although it displays slightly lower selectivity compared to pyridinic-N. Graphitic nitrogen, which substitutes carbon atoms within the graphene planes, integrates into the π-conjugated system and increases electron conductivity. This configuration promotes delocalized charge transfer and moderate Lewis basicity, supporting physisorption-dominated mechanisms through electrostatic interactions and weak hydrogen bonding. Graphitic-N is often associated with improved CO_2_ desorption behavior, contributing to better cyclic stability. Collectively, the synergistic distribution of these nitrogen functionalities modulates surface polarity, electron density, and adsorption site accessibility. However, higher adsorption energy can hinder the diffusion of CO_2_ molecules and slow down the adsorption kinetics. Also, determining the optimal pore size and material properties is critical for effective CO_2_ capture, considering factors such as pressure, temperature, and multi-gas separation scenarios (H_2_O, CO, SO_2_, NO_x_, O_2_, N_2_) [11,57].

The Qiao group investigated the effects of pore structure and surface properties on carbon aerogels, with an isosteric heat of adsorption (Qst) of 24–25 kJ mol^−1^ [58]. These aerogels were carbonized at four different temperatures—600, 700, 800, and 900 °C—resulting in surface area/micropore volumes of 686/0.13, 748/0.13, 1235/0.31, and 888/0.18 m^2^ g^−1^/cm^3^ g^−1^, respectively. CO_2_ uptake at a low pressure (1 bar) at 0 °C followed a trend aligned with the increase or decrease in surface area and pore volume: values of 63, 74, 93, and 81 cm^3^ g^−1^ were obtained for aerogels carbonized at 600, 700, 800, and 900 °C, respectively. As the temperature increased to 25 °C, the adsorption capacity decreased to 43–58 cm^3^ g^−1^, with a similar trend, though the differences in CO_2_ capture between the carbonization temperatures narrowed significantly. This suggests that micropores are highly effective for CO_2_ capture at low pressures and temperatures, but that their role diminishes as temperature rises. However, the study did not specify which pore sizes are most effective at higher temperatures. In humid conditions, all carbonized aerogels exhibited similar adsorption capacities of 11–14 cm^3^ g^−1^ at 50 °C, indicating that micropores are less effective under moisture. This could be due to the fact that the formation of water vapor, which has a smaller molecular size and higher polarity than CO_2_, tends to preferentially occupy these narrow pores, effectively blocking access for CO_2_ and leading to a significant reduction in adsorption capacity. One widely adopted strategy to mitigate this limitation involves hydrophobic surface modification. By functionalizing the carbon surface with non-polar or fluorinated groups, the affinity for water molecules is reduced, thereby preserving the CO_2_ adsorption performance. This can be achieved through post-synthesis treatments using silanes and alkyl chains, or by enhancing graphitic content, which inherently possesses hydrophobic characteristics. The selectivity of CO_2_ over N_2_ at 0 °C was much higher for aerogels carbonized at 600 °C (60–90) compared to those carbonized at 700, 800, and 900 °C (40–70) across a pressure range of 0–1 bar. This indicates that a higher volume ratio of micropores to mesopores is critical for gas separation, as micropores accommodate the smaller kinetic diameter of CO_2_ (0.33 nm), while mesopores are more favorable for the larger kinetic diameter of N_2_ (0.36 nm) [58]. It is also crucial to evaluate solid adsorbents for the temperature swing capture processes under humid conditions, as the working capacity of most solid sorbents typically occurs at 40–60 °C and 0.15 bar, with regeneration taking place at temperatures above 100 °C and 0.8 bar [59]. In contrast, H_2_O adsorption tends to occur at 40–60 °C and 0.06 bar, with regeneration at over 100 °C and pressures below 0.3 bar. Analyzing these factors provides a clearer understanding of the operational requirements for CO_2_ capture applications.

Kanoh’s group developed a carbon aerogel impregnated with potassium carbonate (CA–KC), specifically for CO_2_ capture under moist conditions. Theoretically, alkali metal carbonates capture up to 7.2 mmol g^−1^ of CO_2_ through a reaction—M_2_CO_3_(s) + H_2_O(g) + CO_2_(g) ↔ 2MHCO_3_(s)—in the presence of moisture [60]. The carbon aerogels (CAs) were synthesized by pyrolyzing dried organic aerogels, forming a vitreous monolith with interconnected micro- and mesopores. Nanocrystals of K_2_CO_3_ loaded onto the CAs demonstrated a lower regeneration temperature (150 °C) compared to other potassium-based sorbents. Among the carbon aerogels with different pore sizes (7, 16 and 18 nm), 7CA-KC, i.e., CA with 7 nm pore, exhibited a total CO_2_ capture capacity of 2.68 mmol g^−1^, with the K_2_CO_3_ contributing a CO_2_ capture capacity of 14.5 mmol g^−1^ [60].

Research indicates that micropores smaller than 0.8 nm are particularly effective for CO_2_ capture under low-pressure or direct air capture (DAC) conditions, given CO_2_’s kinetic diameter of 0.33 nm. Pores larger than 0.8 nm and mesopores in the 2–50 nm range are generally less favorable for CO_2_ adsorption under atmospheric conditions [2,11]. To optimize pore formation, various chemical activation agents, such as KOH, NaOH, H_2_PO_4_, CaCl_2_, and HCl, as well as salt templating agents like NaCl, ZnCl_2_, LiBr, KBr, LiI, and KI or gas templating agents like CO_2_, H_2_O, CO, CH_4_, NO, and NH_3_, are commonly used [2,11,61]. Almahdi et al. synthesized N-enriched carbon aerogels (N-CA) from polybenzoxazine, which were crosslinked with graphene oxide–chitosan aerogels [62]. The molecular structure of benzoxazine precursors showed a greater impact towards CO_2_ adsorption. The N-CA obtained from the main chain-type benzoxazine polymer [MCBP (BA-TEPA)], synthesized using BA-a (Bisphenol-A) and TEPA (tetraethylenepentamine), was found to be more effective in terms of CO_2_ adsorption than SLTB (4HBA-t403), i.e., star-like telechelic benzoxazine. The chemical activation of polybenzoxazine with KOH produced aerogels with a high surface area of 1218 m^2^ g^−1^, a pore volume of 0.75 cm^3^ g^−1^, and average pore diameters of 0.87–1.13 nm. These aerogels demonstrated a CO_2_ capture capacity of 7.3 mmol g^−1^ at 25 °C and 1 bar [62]. In related work by the same group, carbon aerogels synthesized by reinforcing montmorillonite into a chitosan-polybenzoxazine framework (MMT-CTS-PBZ) and carbonization at 800 °C exhibited pore sizes ranging from 2 to 7 nm, achieving a CO_2_ adsorption capacity of 5.7 mmol g^−1^ at 25 °C and 1 bar [63]. It is suggested that incorporating chemical activation, salt templating, or physical activation could effectively guide the formation of pores smaller than 1 nm, thereby enhancing CO_2_ capture efficiency under low-pressure conditions.

Cellulose-based carbon aerogels, recognized as third-generation aerogels, offer a range of multifunctional properties, including mechanical strength, thermal and electrical conductivity, lightweight structure, high surface area, enhanced durability, and customizable chemical reactivity. These advanced materials are synthesized using either supercritical drying or ambient-pressure drying techniques. Additionally, third-generation aerogels exhibit biocompatibility, biodegradability, non-toxicity, and substantial sorption capabilities [51,64]. However, cellulose-based aerogels are non-polar and have limited affinity for CO_2_. To address this, the Fu group developed nitrogen-containing functional groups in cellulose-based carbon aerogels through an integrated process of carbonization, activation, and doping [65]. NaOH was used for pore formation (~0.8 nm), carbonization controlled the microstructure, and urea introduced nitrogen-functional groups (pyridinic N, pyrrolic N, and graphitic N). The results showed that the carbon aerogel CNCA-750, which had a well-defined porous structure (total pore volume = 1.081 cm^3^ g^−1^) and rich nitrogen functional groups, achieved a CO_2_ capture capacity of 3.6 mmol g^−1^ and a CO_2_/N_2_ selectivity of 19, under conditions of 15% CO_2_ and 85% N_2_ by volume [65].

**Figure 8:**
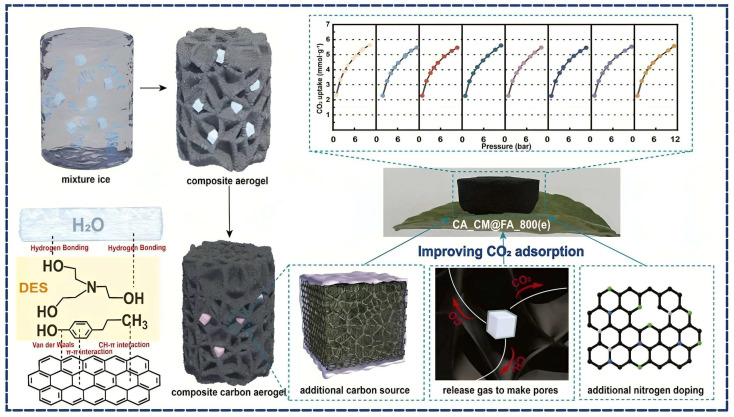
Schematic illustration of strategy for enhancing CO_2_ adsorption in hierarchical porous carbon aerogels through pyrolysis of deep eutectic solvent-encapsulated carbon precursor. Reproduced with permission. Copyright 2025, Elsevier Ltd. [66].

Ma et al. developed a hierarchically porous carbon material derived from metal–organic frameworks (MOFs), referred to as CM, which integrates micro-to-macroporous architectures (Figure 8) [66]. To further enhance pore development, furfuryl alcohol (FA) was introduced into the cavities of the MOF. Acting as a soft template and gas precursor, FA decomposes at elevated temperatures (~800 °C), releasing gases such as H_2_, CO_2_, CO, and CH_4_. These gaseous byproducts serve as pore-forming agents, significantly increasing the pore volume and distribution across multiple scales in the carbon framework. To prevent FA leakage during the synthesis process, a deep eutectic solvent (DES) composed of 4-propylphenol and triethanolamine was employed to encapsulate FA within the MOF cavities. In addition to its encapsulation role, the DES also functioned as a nitrogen source, incorporating pyridinic and graphitic nitrogen functionalities into the carbon structure. These nitrogen species play a crucial role in enhancing CO_2_ adsorption through improved surface polarity and chemical affinity [66].

The resulting carbon aerogel exhibited an exceptionally high specific surface area of 1477 m^2^ g^−1^ and a CO_2_ adsorption capacity of 5.7 mmol g^−1^ under simulated conditions (15% CO_2_/85% N_2_ at 25 °C and 1 bar). It also demonstrated a CO_2_/N_2_ selectivity of 37, representing a 358-fold enhancement over the pristine MOF-derived carbon (CM) without FA or DES modification. Additionally, the composite retained 95% of its CO_2_ uptake capacity after eight consecutive adsorption–desorption cycles, indicating excellent structural and functional stability. This strategy highlights the synergistic role of gas-templated soft templating and nitrogen-doping via DES in engineering advanced porous carbons for efficient and selective CO_2_ capture [66].

### 3.4. Synthesis Approach

The sol–gel approach enables the production of carbon aerogels with uniformly distributed nanopores and high specific surface areas. This method typically involves polycondensation reactions between resorcinol and formaldehyde (RF) precursors, resulting in a wet gel that is subsequently dried and carbonized. While effective in tailoring porosity and mechanical integrity, conventional sol–gel methods often rely on supercritical or freeze-drying techniques, which are complex and cost-intensive [67,68]. To overcome these limitations, the Bhatnagar group developed a cost-effective ambient-pressure drying method for synthesizing RF-based carbon aerogels. Using a resorcinol-to-formaldehyde ratio of 1:2 and triethylamine (TEA) as a catalyst with a resorcinol-to-catalyst (R/C) ratio of 1000, they achieved rapid gelation and minimized shrinkage (~0%) during drying. The resulting aerogels displayed a high surface area of 512.12 m^2^ g^−1^, a small pore size of 1.91 nm, and an impressive CO_2_ capture capacity of 6.7 mmol g^−1^ at 40 bar and 25 °C [69]. Similarly, the Mokaya group produced RF-based carbon aerogels via a subcritical drying method, followed by carbonization at 1050 °C under nitrogen and activation with KOH. By varying the activation temperature (600, 700, and 800 °C) and the KOH-to-carbon ratio (2, 4, and 5), they were able to increase the micropore volume while maintaining pore size. These aerogels exhibited CO_2_ adsorption capacities ranging from 2.7 to 3.0 mmol g^−1^ at 25 °C and 1 bar [70]. RF-derived aerogels are known for their tunable pore structure and high mechanical strength, which can be customized by adjusting synthesis parameters such as the R/C ratio and monomer concentration. These materials have demonstrated broad CO_2_ uptake capacities (2–6 mmol g^−1^), making them strong candidates for scalable CO_2_ capture applications [71,72].

**Figure 9:**
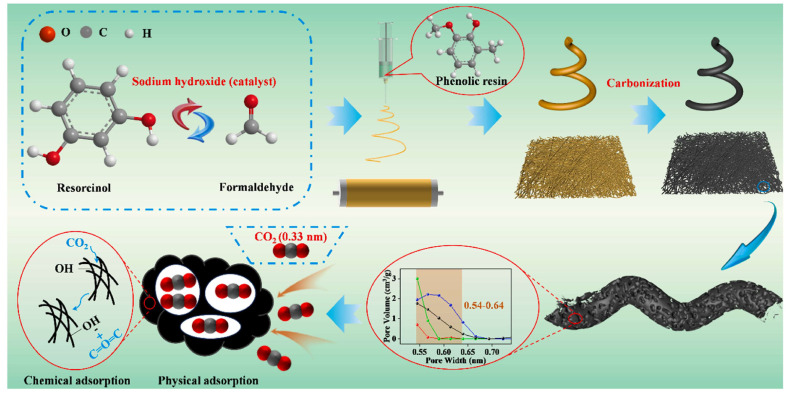
Schematics of preparation of carbon aerogels fibers (CAFs). Reproduced with permission. Copyright 2023, Elsevier Ltd. [73].

Electrospinning is an alternative strategy for fabricating carbon aerogels with controlled three-dimensional architectures. This technique involves applying a high-voltage electric field to a viscoelastic polymer solution, which forms ultrafine nanofibers. The Yi group applied this method to resorcinol–formaldehyde solutions to create carbon aerogel fibers (CAFs), followed by high-temperature carbonization. These fibers offered high surface areas (1188.63 m^2^ g^−1^), high aspect ratios, and approximately 15% oxygen-containing functional groups (Figure 9) [73]. Carbonization temperature significantly influenced pore development, particularly in the 0.54–0.64 nm range. Increasing the temperature from 600 °C to 800 °C resulted in a rise in pore volume from 0.01 cm^3^ g^−1^ to 0.20 cm^3^ g^−1^, but further heating to 900 °C reduced the pore volume to 0.11 cm^3^ g^−1^, likely due to pore collapse. These CAFs achieved a CO_2_ adsorption capacity of 4.25 mmol g^−1^ at 800 °C and one of 3.73 mmol g^−1^ at 900 °C (0 °C, 1 bar), highlighting the effectiveness of ultra-micropores (0.5–0.8 nm) for low-pressure CO_2_ capture. While functional groups may contribute to adsorption, the consistent oxygen content across samples suggests ultra-micropores play a dominant role [73].

Template-based methods, particularly those involving soft or hard templating strategies, enable the synthesis of hierarchical carbon aerogels with multi-modal porosity. The Oksman group developed an efficient approach using kraft lignin and TEMPO-oxidized cellulose nanofibers (TOCNFs), combined through ice templating and carbonization at 1000 °C [74]. During the unidirectional freezing step, aligned ice crystals formed an anisotropic macroporous structure that remained intact after freeze-drying and carbonization. The resultant aerogels featured hierarchical macro-, meso-, and micropores, with a surface area of 1101 m^2^ g^−1^ and a porosity of 93.4%. This hierarchical design enhanced CO_2_ diffusion and adsorption, achieving a CO_2_ capture capacity of 5.2 mmol g^−1^ at 0 °C and 1 bar. The ice templating process offers a simple, scalable pathway to produce advanced porous carbons without the complexity and cost associated with conventional template replication methods [74].

### 3.5. Aerogel-Assisted Photothermal CO_2_ Desorption for Regenerative Capture Systems

**Figure 10:**
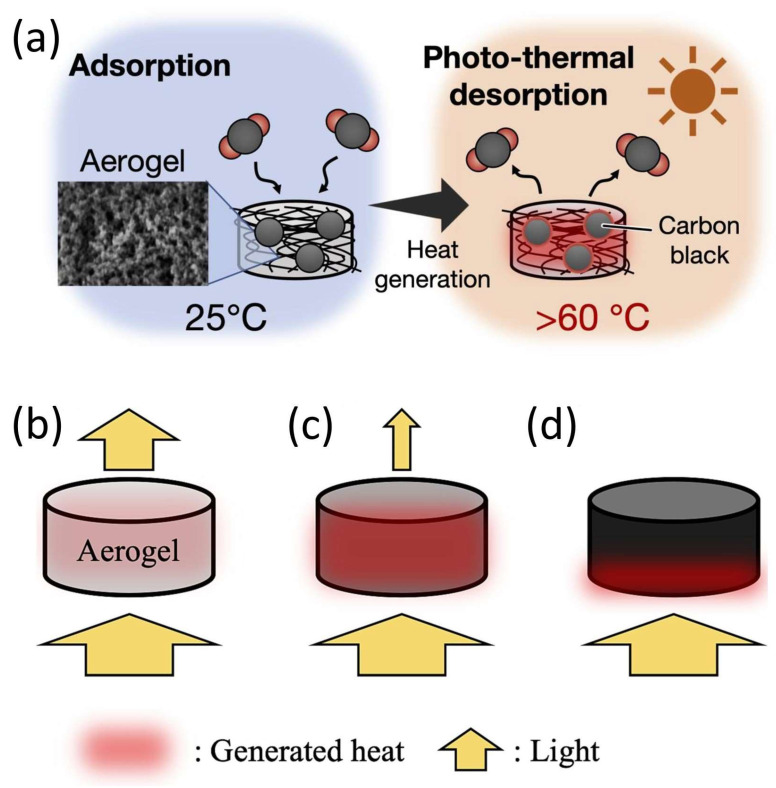
(**a**) Schematics of DAC by aerogel and photo-thermal desorption, and carbon black concentration to generated heat (**b**) small amount, (**c**) optimal amount, and (**d**) excess amount. Reproduced with permission. Copyright 2024, Elsevier B.V. [79].

The direct air capture (DAC) approach is highly suitable for on-site CO_2_ capture, and is particularly valuable for local-scale applications. In DAC processes, aqueous solvents such as amines, calcium hydroxide, and sodium hydroxide are commonly used. Strong bases can rapidly absorb CO_2_, but CO_2_ desorption or solvent regeneration typically requires substantial heat input [11,75]. To address this, photothermal materials have been integrated into absorption–desorption systems to utilize solar energy for heat generation [76]. Nguyen et al. developed carbon nanoparticles dispersed in an amine solvent for CO_2_ absorption, which can then be regenerated through light irradiation [77]. Another approach involves a transparent flow reactor containing amine solvent and photothermal materials that efficiently convert light into heat, facilitating solvent regeneration [78]. However, the use of amine solvents presents challenges, including corrosion, degradation (oxidation and thermal), amine loss, and high energy demands for regeneration [9,57]. An alternative amine-based solid adsorbent method offers advantages, such as lower temperature requirements and the potential for small-scale, scalable DAC units. The Shimoyama group developed an amine-modified silica–carbon aerogel (AmSiC) capable of absorbing solar energy to reach temperatures of 70 °C for CO_2_ desorption, achieving an efficiency of 80% (Figure 10) [79]. Studies show that AmSiC aerogel, which has a pore volume of 0.8 cm^3^ g^−1^ and is dried through supercritical CO_2_ drying, exhibits a CO_2_ desorption rate 60 times higher than that of xerogel (which has undetectable pore volume) dried via thermal evaporation. This significant enhancement in desorption is attributed to the superior thermal insulation properties of the porous structure in AmSiC aerogels [79].

### 3.6. Multifunctional Aerogels for Integrated CO_2_ Capture and Conversion

Recent advancements in aerogel materials have enabled the development of multifunctional systems that integrate CO_2_ capture with catalytic conversion, addressing both separation and utilization challenges. These aerogels leverage hierarchical porosity, tunable surface chemistry, and structural robustness to enhance adsorption capacity while facilitating chemical transformation of captured CO_2_ [80,81,82].

**Figure 11:**
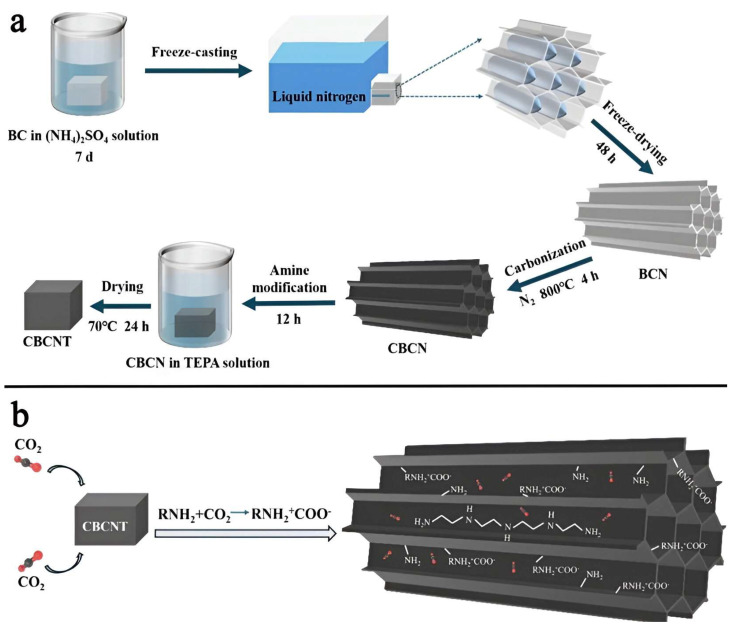
(**a**) Schematics of CBCNT preparation, and (**b**) schematics of the CO_2_ adsorption on modified CBCNT. Reproduced with permission. Copyright 2024, Elsevier Ltd. [86].

Bacterial cellulose (BC), a renewable biopolymer, offers a promising route for fabricating carbon nanofiber aerogels due to its highly interconnected 3D network of nanofibers with high aspect ratios. This robust nanofiber framework provides mechanical stability under stress; however, direct carbonization often yields disordered pore structures that hinder effective CO_2_ diffusion and adsorption [83,84]. To overcome these limitations, freeze-drying and freeze-casting techniques have been employed to create aligned porous architectures, where temperature gradients guide the formation of directional ice crystals, sandwiching BC nanofibers. Upon ice sublimation, a continuous nanofiber network is retained. Yet, carbonizing these structures typically results in thin, fragile aerogels with compromised mechanical integrity. To stabilize the architecture during high-temperature processing, a salt-templating strategy using ammonium sulfate ((NH_4_)_2_SO_4_) was introduced. This approach interlocks with the nanofiber network during calcination, preserving the honeycomb-like morphology and enhancing elasticity. Additionally, (NH_4_)_2_SO_4_ acts as a nitrogen source, enriching the carbon framework with N-functional groups that boost CO_2_ affinity. Mei et al. further enhanced performances by incorporating tetraethylenepentamine (TEPA), leading to three material variants: CBC (carbonized BC), CBCN ((NH_4_)_2_SO_4_-modified), and CBCNT (with both (NH_4_)_2_SO_4_ and TEPA). CBCNT exhibited the highest CO_2_ uptake at 0 °C and 1 bar (4.88 mmol g^−1^), demonstrating the synergistic effects of N-doping and amine functionalization (Figure 11) [85,86]. Despite these improvements, this study lacks a detailed mechanistic understanding of the relative contributions of surface chemistry versus pore architecture in driving adsorption. Future research should aim to deconvolute these factors to guide the rational design of high-performance sorbents.

**Figure 12:**
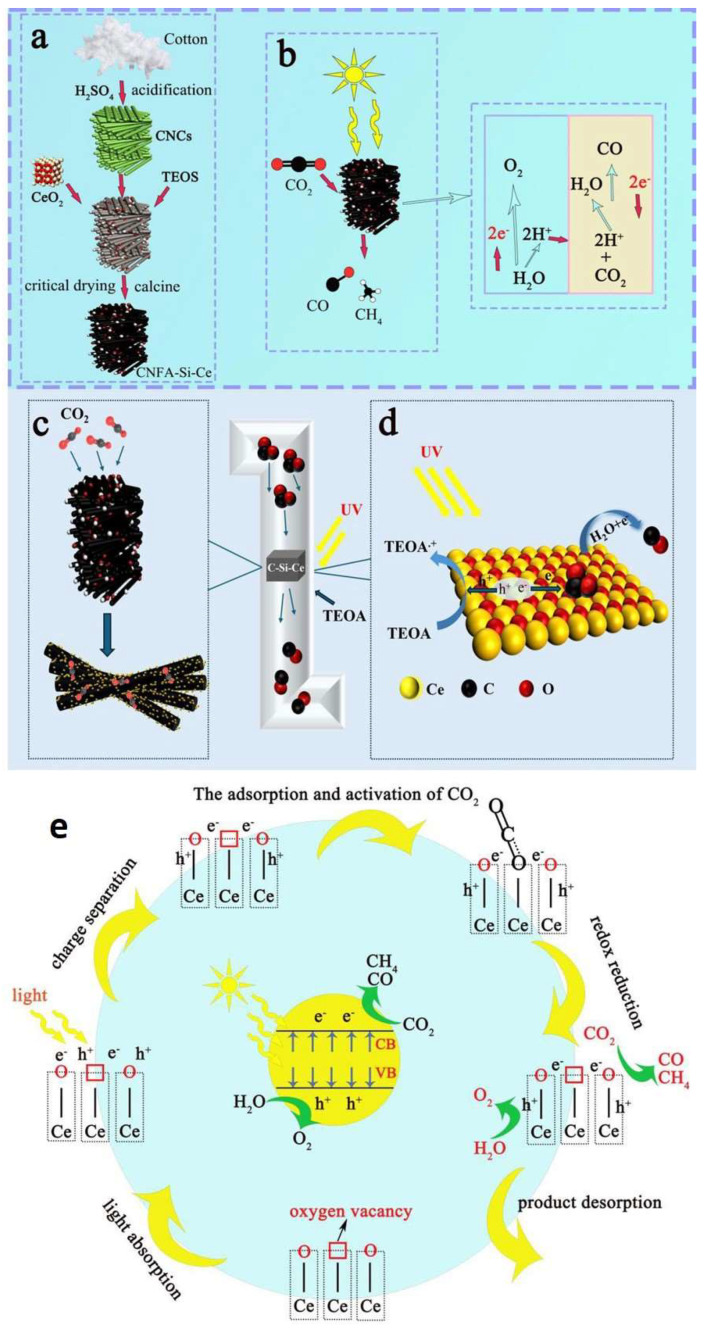
(**a**,**b**) Illustrations depicting synthesis of bifunctional materials and their simulated conversion process under light exposure, (**c**,**d**) representation of CO_2_ capture and subsequent transformation using dual-functional materials within an integrated system, and (**e**) mechanism of photocatalytic CO_2_ reduction at oxygen vacancy sites on cerium oxide. Reproduced with permission. Copyright 2024, Elsevier Inc. [92].

To advance beyond capture alone, integration with catalytic CO_2_ conversion was explored. Conventional reduction processes often suffer from energy-intensive requirements due to CO_2_’s thermodynamic stability. Photocatalysis presents a sustainable alternative by utilizing solar energy to activate and convert CO_2_ under mild conditions [87,88,89,90,91]. Rong et al. developed a silica–cerium-doped cellulose-derived carbon aerogel (CNFA–Si–Ce) by combining cellulose nanocrystals with tetraethyl orthosilicate (TEOS) and cerium nitrate, followed by supercritical drying and calcination (Figure 12) [92]. The resulting material featured a hierarchically porous structure that enhanced both CO_2_ diffusion and adsorption. Cerium, known for its photocatalytic properties, was strategically incorporated to engineer electronic vacancies in the aerogel matrix. These vacancies—particularly oxygen vacancies—serve as active sites that lower the activation barrier for CO_2_ adsorption and facilitate its reduction. The CNFA–Si–Ce aerogel exhibited a CO_2_ uptake of 3.18 mmol g^−1^ at 25 °C and 1 bar. Under simulated solar light, the catalyst achieved a CO production rate of 59.5 µmol h^−1^ and an electron consumption rate of 119 µmol h^−1^ g^−1^, underscoring its efficient conversion performance [93,94,95,96]. This integrated system exemplifies the potential of multifunctional aerogels that unify capture and conversion processes. By combining tailored pore architecture, surface functionalization, and catalytic elements, such aerogels offer a low-energy, scalable platform for CO_2_ mitigation and valorization.

### 3.7. Artificial Intelligence for Aerogels Design

The synthesis of carbon aerogels typically follows a standard process; however, the final properties of the materials are highly sensitive to a wide range of parameters and processing conditions. These variables—spanning precursor composition, activation methods, and carbonization conditions—result in multidimensional property outputs that are difficult to predict using conventional experimental approaches. In this context, machine learning (ML) has emerged as a powerful tool to establish quantitative relationships between synthesis parameters and the resulting material properties [97]. Data-driven models enable the abstraction of complex synthesis–property relationships, providing predictive insights and guiding the rational design of carbon aerogels for specific applications [70].

The synthesis of carbon aerogels often involves diverse chemical precursors and a variety of processing conditions, including the mass ratio of activation agents to precursors, carbonization temperature and duration, heating rate, gas flow rate and type, reactor volume, and the type of activating agent—either chemical (e.g., KOH, H_3_PO_4_, ZnCl_2_) or physical (e.g., steam, CO_2_) [97,98]. These variables collectively determine critical properties such as surface area, pore size distribution, and structural integrity. Machine learning models trained on experimental datasets have demonstrated the ability to predict these properties with high accuracy. Notably, the hydrogen content in precursor materials has been shown to influence outcomes when using physical activation methods, while the mass ratio of chemical activating agents has a strong effect on the specific surface area [99,100]. Thus, integrating machine learning with experimental design holds significant promise for optimizing carbon aerogel synthesis and tailoring their functionalityto targeted applications.

## 4. Summary and Future Research Strategies

In conclusion, carbon-based aerogels have shown promising CO_2_ capture performance, with adsorption capacities ranging from 2 to 7 mmol g^−1^ at low pressures (Table 1). This was largely achieved through the careful tailoring of textural properties and selective functional group introduction. Recent research underscores the importance of microstructural and textural characteristics in optimizing carbon aerogels for CO_2_ capture, supported by both experimental evidence and theoretical models. Critical properties, including pore size distribution (ultra-, super-, micro-, meso-, and macropores), surface area, and surface chemistry, significantly influence adsorption capacities. For example, micropores under 2 nm align with CO_2_’s kinetic diameter (0.33 nm), promoting effective and selective CO_2_ adsorption, particularly at low pressures. While mesopores can be advantageous for high-pressure applications, they may also favor the adsorption of N_2_ (0.36 nm), posing a selectivity challenge for CO_2_ capture.

Considering the properties of the carbon aerogels (regardless of the carbon origin), the pore size and pore volume of the carbon aerogel play an important role, in spite of increasing or decreasing surface area. In particular, micropore size (between 0.8 and 2.0 nm) and micropore volume (between 0.31 and 0.75 cm^3^ g^−1^) are effective in terms of CO_2_ adsorption under normal conditions. Conversely, a higher volume ratio of micropores to mesopores is effective under elevated conditions and for CO_2_/N_2_ selectivity. In addition to this, the synthesis strategy pertaining to the use of conductive support techniques such as rGO; N-doped carbon aerogels from polybenzoxazine; electrospinning; varying precursor ratios; pore formers; chemical activating agents such as KOH and K_2_CO_3_; and a suitable carbonization temperature (max of 800 °C) favors an increase in CO_2_ adsorption capacity from 0 to 50 °C and from 1 to 40 bar.

Surface functional groups further enhance CO_2_ uptake by facilitating interactions like hydrogen bonding, electrostatic attraction, and acid–base interactions. Understanding the functional characteristics, such as the roles of pyridinic, pyrrolic, or graphitic nitrogen groups, is essential for rational design, although a unified understanding of which interaction modes are most effective for CO_2_ capture remains a research objective. To date, evaluations of CO_2_ capture efficiency often lack a unified strategy, frequently neglecting to address varying conditions such as moisture swing adsorption or temperature swing adsorption. This oversight complicates fair comparisons across different material design approaches. Establishing clear operational parameters is essential for accurately comparing materials under conditions that mimic real-world applications. Categorizing capture modes based on these operational perspectives will not only facilitate further development but also enhance our understanding of performance outcomes in practical, real-world scenarios.

Carbon aerogels, in particular, offer the advantage of a fixed-shape adsorbent suited for practical applications. However, standardized methods for shaping aerogel-based adsorbents must be established, as improper preparation techniques—such as attempting to convert powdered materials with optimal properties into fixed shapes—can result in the degradation of textural, microstructural, and functional properties. Developing a pre-pelletization approach during synthesis, along with a tailored property-tuning strategy, is crucial to maintaining performance in a fixed-shape format. This could address the current challenges of converting fine materials into final adsorbent forms, which are often produced using binders or high-pressure compression, leading to substandard products. An optimized fixed-shape strategy will enable more consistent performance in real-world applications.

The experimental design and synthesis of aerogel materials, including their preparation, storage, required equipment, and fabrication processes, are often cost-intensive due to prolonged synthesis times and extensive post-processing requirements. Moreover, the synthesis of these materials typically demands precise control over sensitive experimental conditions—such as temperature—and relies heavily on the expertise of researchers, making the process both resource- and labor-intensive. In this context, data-driven approaches employing artificial intelligence (AI), particularly machine learning techniques such as neural networks, offer promising alternatives. These approaches enable the extraction of valuable insights from existing datasets, deliver high computational accuracy and predictive performance, and allow for the efficient use of stored data for forecasting purposes. By accelerating the development of effective synthesis frameworks, AI-driven methodologies hold significant potential to reduce, or even eliminate, the financial and operational burdens associated with traditional experimental protocols, thereby facilitating the rational design and optimization of novel aerogel materials.

## Figures and Tables

**Table 1:** Comparisons of CO_2_ sorption performance of various carbon aerogels.

Materials	Performances
N-doped C aerogels [54]	6.1 mmol g^−1^ (0 °C, 1 bar), 33.1 mmol g^−1^ (50 °C, 30 bar), selectivity 47.8 (CO_2_/N_2_) at ambient pressure
MgAl-MMO/rGO aerogel [56]	2.4 mmol g^−1^ (300 °C, 8 bar)
Microemulsion templated C aerogel [58]	63 mmol g^−1^ (0 °C, 1 bar), selectivity (15%CO_2_/N_2_)
C aerogels-K_2_CO_3_ [60]	Total CO_2_ capture 2.7 mmol g^−1^, CO_2_ capture capacity by K_2_CO_3_ 14.5 mmol g^−1^
Hierarchical C aerogels [66]	5.7 mmol g^−1^ (0 °C, 1 bar), selectivity (15%CO_2_/N_2_)
C aerogel based on formaldehyde /resorcinol/triethyl amine [69]	6.7 mmol g^−1^ at 25 °C, 40 bar
C aerogel based on formaldehyde/melamine [101]	2.2 mmol g^−1^ at 25 °C, 1 bar
Microporous activated C aerogel [102]	3 mmol g^−1^ at 25 °C, 1 bar
C aerogel based on chitosan/polybenzoxazine [62]	7.3 mmol g^−1^ at 25 °C, 1 bar
Cellulose based N-doped C aerogel [65]	3.6 mmol g^−1^ (20 °C, 1 bar), selectivity (15%CO_2_/85N_2_)
Lignin/TOCNF C aerogel [74]	5.2 mmol g^−1^ (0 °C, 1 bar)
C aerogel based on chitosan/polybenzoxazine [63]	5.7 mmol g^−1^ at 25 °C, 1 bar
N-doped C aerogel [103]	118 mg g^−1^ (2.68 mmol g^−1^) at 25 °C, 1 bar
C aerogel based on formaldehyde/resorcinol [104]	83.7 cm^3^ g^−1^ (3.73 mmol g^−1^) at (0 °C), 56.5 (25 °C), 18.5 (50 °C) at 1 bar
N-doped C aerogel [105]	4.8 mmol g^−1^ at 1 bar
C aerogel fiber [69]	4.2 mmol g^−1^ at 0 °C, 1 bar
Cellulose based C aerogel [47]	15 mmol g^−1^ at 25 °C, 30 bar
Monolithic C aerogel [48]	4.5 mmol g^−1^ at 25 °C, 1 bar
Cellulose based hierarchical C aerogels [51]	3.4 mmol g^−1^ at 25 °C, 1 bar
C aerogel [70]	3 mmol g^−1^ at 25 °C, 1 bar
Bacterial cellulose C naofiber aerogel/TEPA [86]	4.88 mmol g^−1^ at 0 °C, 1 bar, selectivity (15CO_2_/85N_2_)
Ce-doped porous C aerogel [92]	3.18 mmol g^−1^ at 0 °C, 1 bar

C: carbon; N-doped: nitrogen-doped; MMO/rGO: mixed metal oxides/reduced graphene oxide; TEPA: tetraethylenepentamine; TOCNF: TEMPO (2,2,6,6-tetramethylpiperidine-1-oxyl radical) oxidized cellulose nanofibers.

## Data Availability

No new data were created or analyzed in this study.

## References

[1] Jasmine K. The Concentration of CO_2_ in the Atmosphere Could Reach 550 Parts per Million by 2050. A 32 % Increase Compared to 2021. The World Counts • Impact through Awareness. https://www.theworldcounts.com/challenges/global-warming/CO2-concentration.

[2] Bari G.A.K.M.R., Kang H.-J., Lee T.-G., Hwang H.J., An B.-H., Seo H.-W., Ko C.H., Hong W.H., Jun Y.-S. (2023). Dual-templating-derived porous carbons for low-pressure CO_2_ capture. Carbon. Lett..

[3] Brethomé F.M., Williams N.J., Seipp C.A., Kidder M.K., Custelcean R. (2018). Direct air capture of CO_2_ via aqueous-phase absorption and crystalline-phase release using concentrated solar power. Nat. Energy.

[4] Clifford C. Carbon Capture Challenges Are Not Deterring Investor at Bill Gates’ Firm. https://www.cnbc.com/2022/05/07/what-is-carbon-capture-eric-toone-investor-at-gates-firm-explains.html?_hsenc=p2ANqtz-_lvXFc1Ucw6m2b_evUgXwAL1FyrCI2aZw4fZftQ19CbbEwLpZyCN1jgpc-xgGJUq4z78fu.

[5] Buis A. (2019). Examining the Viability of Planting Trees to Help Mitigate Climate Change. NASA’s Jet. Propuls. Lab..

[6] Islam M., Han D., Bari G.A.K.M.R., Nam K. (2024). Electrochemical Storage Behavior of a High-Capacity Mg-Doped P2-Type Na2/3Fe1−yMnyO2 Cathode Material Synthesized by a Sol–Gel Method. Gels.

[7] Park S., Kim S., Bari G.A.K.M.R., Jeong J. (2024). Fundamental Understanding of Marine Applications of Molten Salt Reactors: Progress, Case Studies, and Safety. J. Mar. Sci. Eng. Sci. Eng..

[8] Bari G.A.K.M.R., Jeong J.-H. (2024). Comprehensive Insight and Advancements in Material Design for Electrocatalytic Ammonia Production Technologies: An Alternative Clean Energy. Int. J. Energy Res..

[9] Bari G.A.K.M.R., Jeong J.-H. (2024). Comprehensive Insights and Advancements in Gel Catalysts for Electrochemical Energy Conversion. Gels.

[10] Khan T.T., Bari G.A.K.M.R., Kang H.-J., Lee T.-G., Park J.-W., Hwang H.J., Hossain S.M., Mun J.S., Suzuki N., Fujishima A. (2021). Synthesis of N-doped TiO2 for efficient photocatalytic degradation of atmospheric NOx. Catalysts.

[11] Bari G.A.K.M.R., Jeong J.-H. (2023). Porous Carbon for CO_2_ Capture Technology: Unveiling Fundamentals and Innovations. Surfaces.

[12] Liu C. (2024). Multi-stage solvent circulation absorption enhancement: System optimization for energy-saving CO_2_ capture. Sep. Purif. Technol..

[13] Li Q., Huang X., Li N., Qi T., Wang R., Wang L., An S. (2024). Energy-efficient biphasic solvents for industrial CO_2_ capture: Absorption mechanism and stability characteristics. Energy.

[14] Buvik V., Høisæter K.K., Vevelstad S.J., Knuutila H.K. (2021). A review of degradation and emissions in post-combustion CO_2_ capture pilot plants. Int. J. Greenh. Gas Control..

[15] Fytianos G., Ucar S., Grimstvedt A., Hyldbakk A., Svendsen H.F., Knuutila H.K. (2016). Corrosion and degradation in MEA based post-combustion CO_2_ capture. Int. J. Greenh. Gas Control.

[16] Bougie F., Iliuta M.C. (2014). Stability of aqueous amine solutions to thermal and oxidative degradation in the absence and the presence of CO_2_. Int. J. Greenh. Gas Control.

[17] Grimstvedt A., Zahlsen K., Vevelstad S.J., Vernstad K., Holten T., Brunsvik A. (2017). Exploration of Degradation Chemistry by Advanced Analytical Methodology. Energy Procedia.

[18] Singh G., Lee J., Karakoti A., Bahadur R., Yi J., Zhao D., AlBahily K., Vinu A. (2020). Emerging trends in porous materials for CO_2_ capture and conversion. Chem. Soc. Rev..

[19] Wang Q., Zhu X., Liu Y., Fang Y., Zhou X., Bao J. (2018). Rice husk-derived hard carbons as high-performance anode materials for sodium-ion batteries. Carbon.

[20] Xu K., Li Y., Xiong J., Ou X., Su W., Zhong G., Yang C. (2018). Activated Amorphous Carbon With High-Porosity Derived From Camellia Pollen Grains as Anode Materials for Lithium/Sodium Ion Batteries. Front. Chem..

[21] Rehman A., Heo Y.J., Nazir G., Park S.J. (2021). Solvent-free, one-pot synthesis of nitrogen-tailored alkali-activated microporous carbons with an efficient CO_2_ adsorption. Carbon.

[22] Keith D.W., Holmes G., Angelo D.S., Heidel K. (2018). A Process for Capturing CO_2_ from the Atmosphere. Joule.

[23] Sabatino F., Grimm A., Gallucci F., van Sint Annaland M., Kramer G.J., Gazzani M. (2021). A comparative energy and costs assessment and optimization for direct air capture technologies. Joule.

[24] Honda R., Hamasaki A., Miura Y., Hoshino Y. (2021). Thermoresponsive CO_2_ absorbent for various CO_2_ concentrations: Tuning the pK a of ammonium ions for effective carbon capture. Polym. J..

[25] Wang T., Wang X., Hou C., Liu J. (2020). Quaternary functionalized mesoporous adsorbents for ultra-high kinetics of CO_2_ capture from air. Sci. Rep..

[26] Al-Muhtaseb S.A., Ritter J.A. (2003). Preparation and Properties of Resorcinol–Formaldehyde Organic and Carbon Gels. Adv. Funct. Mater..

[27] Salimian S., Zadhoush A., Naeimirad M., Kotek R., Ramakrishna S. (2018). A Review on Aerogel: 3D Nanoporous Structured Fillers in Polymer-Based Nanocomposites. Polym. Compos..

[28] ElKhatat A.M., Al-Muhtaseb S.A. (2011). Advances in Tailoring Resorcinol-Formaldehyde Organic and Carbon Gels. Adv. Mater..

[29] Kang H.J., Huh Y.S., Im W.B., Jun Y.S. (2019). Molecular cooperative assembly-mediated synthesis of ultra-high-performance hard carbon anodes for dual-carbon sodium hybrid capacitors. ACS Nano.

[30] Vilian A., Song J.Y., Lee Y.S., Hwang S.-K., Kim H.J., Jun Y.-S., Huh Y.S., Han Y.-K. (2018). Salt-templated three-dimensional porous carbon for electrochemical determination of gallic acid. Biosens. Bioelectron..

[31] Yang W., Yang W., Zou R., Huang Y., Lai H., Chen Z. (2023). Cellulose nanofiber-derived carbon aerogel for advanced room-temperature sodium–sulfur batteries. Carbon Energy.

[32] Ye J., Li X., Gao W., Zhu Y. (2020). In Situ Nitrogen-Doping Carbon Aerogel as an Effective Sulfur Host to Immobilize Polysulfides for High Performance Lithium-Sulfur Battery. ChemistrySelect.

[33] Cai T., Kuang L., Wang C., Jin C., Wang Z., Sun Q. (2019). Cellulose as an Adhesive for the Synthesis of Carbon Aerogel with a 3D Hierarchical Network Structure for Capacitive Energy Storage. ChemElectroChem.

[34] Kamran U., Rhee K.Y., Lee S.Y., Park S.J. (2022). Solvent-free conversion of cucumber peels to N-doped microporous carbons for efficient CO_2_ capture performance. J. Clean. Prod..

[35] Yang I., Jung M., Kim M.S., Choi D., Jung J.C. (2021). Physical and chemical activation mechanisms of carbon materials based on the microdomain model. J. Mater. Chem. A Mater..

[36] Mallikarjuna K., Bari G.A.K.M.R., Vattikuti S.V.P., Kim H. (2020). Synthesis of carbon-doped SnO_2_ nanostructures for visible-light-driven photocatalytic hydrogen production from water splitting. Int. J. Hydrogen Energy.

[37] Mandari K.K., Son N., Pandey S., Kim Y.S., Bari G.A.K.M.R., Kang M. (2020). Nb2O5–SnS2–CdS heteronanostructures as efficient visible-light-harvesting materials for production of H2 under solar light irradiation. J. Alloys Compd.

[38] Bari G.A.K.M.R., Islam M., Jeong J.-H. (2024). Materials Design and Development of Photocatalytic NOx Removal Technology. Metals.

[39] Liu X., Giordano C., Antonietti M. (2014). A facile molten-salt route to graphene synthesis. Small.

[40] Fechler N., Fellinger T.P., Antonietti M. (2013). “Salt templating”: A simple and sustainable pathway toward highly porous functional carbons from ionic liquids. Adv. Mater..

[41] Sonu S.S., Rai N., Chauhan I. (2023). Multifunctional Aerogels: A comprehensive review on types, synthesis and applications of aerogels. J. Solgel Sci. Technol..

[42] Zhang Z., Wang P., Zhang W., Hu X., Zhang X., Gou Z., Xu W., Zheng H., Ding X. (2024). A review: Recent advances in conductive aerogels: Assembly strategies, conductive mechanisms, influencing factors and applications. J. Mater. Sci..

[43] Bari G.A.K.M.R., Jeong J., Barai H.R. (2024). Conductive Gels for Energy Storage, Conversion, and Generation: Materials Design Strategies, Properties, and Applications. Materials.

[44] Kang H.-J., Lee T.-G., Bari G.A.K.M.R., Seo H.-W., Park J.-W., Hwang H.J., An B.-H., Suzuki N., Fujishima A., Kim J.-H. (2021). Sulfuric acid treated G-CN as a precursor to generate high-efficient G-CN for hydrogen evolution from water under visible light irradiation. Catalysts.

[45] Bano S., Negi Y.S. (2017). Studies on cellulose nanocrystals isolated from groundnut shells. Carbohydr. Polym..

[46] Thomas B., Geng S., Sain M., Oksman K. (2021). Hetero-porous, high-surface area green carbon aerogels for the next-generation energy storage applications. Nanomaterials.

[47] Cheng J., Cheng X., Wang Z., Hussain M.B., Wang M. (2023). Multifunctional carbon aerogels from typha orientalis for applications in adsorption: Hydrogen storage, CO_2_ capture and VOCs removal. Energy.

[48] Geng S., Maennlein A., Yu L., Hedlund J., Oksman K. (2021). Monolithic carbon aerogels from bioresources and their application for CO_2_ adsorption. Microporous Mesoporous Mater..

[49] Choi S., Drese J.H., Jones C.W. (2009). Adsorbent materials for carbon dioxide capture from large anthropogenic point sources. ChemSusChem.

[50] Wahby A., Ramos-Fernández J.M., Martínez-Escandell M., Sepúveda-Escribano A., Silvestre-Albero J., Rodríguez-Reinoso F. (2010). High-surface-area carbon molecular sieves for selective CO_2_ adsorption. ChemSusChem.

[51] Zhuo H., Hu Y., Tong X., Zhong L., Peng X., Sun R. (2016). Sustainable hierarchical porous carbon aerogel from cellulose for high-performance supercapacitor and CO2 capture. Ind. Crops Prod..

[52] Cai J., Kimura S., Wada M., Kuga S., Zhang L. (2008). Cellulose aerogels from aqueous alkali hydroxide-urea solution. ChemSusChem.

[53] Hao P., Zhao Z., Tian J., Li H., Sang Y., Yu G., Cai H., Liu H., Wong C.P., Umar A. (2014). Hierarchical porous carbon aerogel derived from bagasse for high performance supercapacitor electrode. Nanoscale.

[54] Li H., Li J., Thomas A. (2019). Ultra-High Surface Area Nitrogen-Doped Carbon Aerogels Derived From a Schiff-Base Porous Organic Polymer Aerogel for CO_2_ Storage and Supercapacitors. Adv. Funct. Mater..

[55] Dong J., Zhu T., Li H., Sun H., Wang Y., Niu L., Wen X., Bai G. (2019). Biotemplate-Assisted Synthesis of Layered Double Oxides Confining Ultrafine Ni Nanoparticles as a Stable Catalyst for Phenol Hydrogenation. Ind. Eng. Chem. Res..

[56] Xia D., Li H., Mannering J., Huang P., Zheng X., Kulak A., Baker D., Iruretagoyena D., Menzel R. (2020). Electrically Heatable Graphene Aerogels as Nanoparticle Supports in Adsorptive Desulfurization and High-Pressure CO_2_ Capture. Adv. Funct. Mater..

[57] Bari G.A.K.M.R., Jeong J.-H. (2024). Potential of Carbon Aerogels in Energy: Design, Characteristics, and Applications. Gels.

[58] Liu Q., Han Y., Qian X., He P., Fei Z., Chen X., Zhang Z., Tang J., Cui M., Qiao X. (2019). CO_2_ Adsorption over Carbon Aerogels: The Effect of Pore and Surface Properties. ChemistrySelect.

[59] Begag P.N.R., Krutka H., Dong W., Mihalcik D., Rhine W., Gould G., Baldic J. (2013). Superhydrophobic amine functionalized aerogels as sorbents for CO_2_ capture. Greenh. Gases Sci. Technol..

[60] Yang G., Luo H., Ohba T., Kanoh H. (2016). CO_2_ Capture by Carbon Aerogel-Potassium Carbonate Nanocomposites. Int. J. Chem. Eng..

[61] Singh G., Ruban A.M., Geng X., Vinu A. (2023). Recognizing the potential of K-salts, apart from KOH, for generating porous carbons using chemical activation. Chem. Eng. J..

[62] Alhwaige A.A., Ishida H., Qutubuddin S.A. (2024). Nitrogen-Enriched Carbon Aerogels Derived from Polybenzoxazine Cross-Linked Graphene Oxide-Chitosan Hybrid Matrix with Superior CO_2_ Capture Performance. ACS Appl. Eng. Mater..

[63] Alhwaige A.A., Ishida H., Qutubuddin S. (2016). Carbon Aerogels with Excellent CO2 Adsorption Capacity Synthesized from Clay-Reinforced Biobased Chitosan-Polybenzoxazine Nanocomposites. ACS Sustain. Chem. Eng..

[64] Miao Y., Pudukudy M., Zhi Y., Miao Y., Shan S., Jia Q., Ni Y. (2020). A facile method for in situ fabrication of silica/cellulose aerogels and their application in CO_2_ capture. Carbohydr. Polym..

[65] Li T., An X., Chen J., Fan L., Fu D. (2024). One-Step Synthesis of Cellulosed-Based Nitrogen-Doped Carbon Aerogel and Its CO2 Adsorption Performance. Energy Fuels.

[66] Ma H., Cui B., Li J., Ju X., Wang D., Yang Z. (2025). Enhancing CO_2_ adsorption in hierarchical carbon aerogels via deep eutectic solvent-encapsulated carbon source pyrolysis. J. Environ. Chem. Eng..

[67] Chai S., Dai X., Wu T., Liu B., Yao H., Yuan Y., Wu Q. (2020). Synthesis of Si/O/C/N Quaternary Composite Aerogels with Micro/Mesoporous Structures and Their Selective Adsorption Property for Volatile Carbonyl Compounds in Cigarette Smoke. Microporous Mesoporous Mater..

[68] Ekabutr P., Ariyathanakul T., Chaiyo S., Niamlang P., Rattanaveeranon S., Chailapakul O., Supaphol P. (2018). Carbonized Electrospun Polyvinylpyrrolidone/Metal Hybrid Nanofiber Composites for Electrochemical Applications. J. Appl. Polym. Sci..

[69] Bhatnagar A., Pandey A.P., Hudson M.S.L., Soni P.K., Verma S.K., Shukla V., Sekkar V., Tripathi M., Srivastava O.N. (2021). Economical Synthesis of Highly Efficient and Tunable Carbon Aerogels for Enhanced Storage of CO_2_ Emitted from Energy Sources. Int. J. Energy Res..

[70] Robertson C., Mokaya R. (2013). Microporous Activated Carbon Aerogels via a Simple Subcritical Drying Route for CO_2_ Capture and Hydrogen Storage. Microporous Mesoporous Mater..

[71] Singh S., Bhatnagar A., Dixit V., Shukla V., Shaz M.A., Sinha A.S.K., Srivastava O.N., Sekkar V. (2016). Synthesis, Characterization and Hydrogen Storage Characteristics of Ambient Pressure Dried Carbon Aerogel. Int. J. Hydrogen Energy.

[72] Kong Y., Shen X., Cui S., Fan M. (2014). Use of Monolithic Silicon Carbide Aerogel as a Reusable Support for Development of Regenerable CO_2_ Adsorbent. RSC Adv..

[73] Wang Y., Tang X., Gao S., Jiang L., Yi Y. (2024). Study of CO_2_ adsorption on carbon aerogel fibers prepared by electrospinning. J. Environ. Manag..

[74] Geng S., Wei J., Jonasson S., Hedlund J., Oksman K. (2020). Multifunctional Carbon Aerogels with Hierarchical Anisotropic Structure Derived from Lignin and Cellulose Nanofibers for CO_2_ Capture and Energy Storage. ACS Appl. Mater. Interfaces.

[75] Sanz-Pérez E.S., Murdock C.R., Didas S.A., Jones C.W. (2016). Direct Capture of CO_2_ from Ambient Air. Chem. Rev..

[76] Gu Y., Mu X., Wang P., Wang X., Liu J., Shi J., Wei A., Tian Y., Zhu G., Xu H. (2020). Integrated photothermal aerogels with ultrahigh-performance solar steam generation. Nano Energy.

[77] Nguyen D.T., Truong R., Lee R., Goetz S.A., Esser-Kahn A.P. (2014). Photothermal release of CO_2_ from capture solutions using nanoparticles. Energy Environ. Sci..

[78] Campbell Z.S., Han S., Marre S., Abolhasani M. (2021). Continuous Flow Solar Desorption of CO_2_ from Aqueous Amines. ACS Sustain. Chem. Eng..

[79] Kataoka T., Orita Y., Shimoyama Y. (2024). Photo-thermal CO_2_ desorption from amine-modified silica / carbon aerogel for direct air capture. Chem. Eng. J..

[80] Wu Z., Chen S., Li J., Wang B., Jin M., Liang Q., Zhang D., Han Z., Deng L., Qu X. (2023). Insights into Hierarchical Structure–Property–Application Relationships of Advanced Bacterial Cellulose Materials. Adv. Funct. Mater..

[81] Bahadur R., Singh G., Li M., Chu D., Yi J., Karakoti A., Vinu A. (2023). BCN Nanostructures Conjugated Nanoporous Carbon with Oxygenated Surface and High Specific Surface Area for Enhanced CO2 Capture and Supercapacitance. Chem. Eng. J..

[82] Titirici M.M., Thomas A., Yu S.H., Müller J.O., Antonietti M. (2007). A Direct Synthesis of Mesoporous Carbons with Bicontinuous Pore Morphology from Crude Plant Material by Hydrothermal Carbonization. Chem. Mater..

[83] Ummah M.S. (2023). 3Dfibrousaerogels from 1Dpolymernanofibersfor Energy and Environmental Applications. J. Mater. Chem. A.

[84] Wang T., Meng X., Li P., Ouyang S., Chang K., Liu G., Mei Z., Ye J. (2014). Photoreduction of CO_2_ over the Well-Crystallized Ordered Mesoporous TiO_2_ with the Confined Space Effect. Nano Energy.

[85] Sharma N.K., Verma C.S., Chariar V.M., Prasad R. (2015). Eco-Friendly Flame-Retardant Treatments for Cellulosic Green Building Materials. Indoor Built Environ..

[86] Bao S., Zheng X., Xu Z., Ji B., Yang Z., Sun W., Mei J., Rong J., Li Z. (2025). Amine-impregnated elastic carbon nanofiber aerogel templated by bacterial cellulose for CO_2_ adsorption and separation. Fuel.

[87] Chen S., Liu J., Zhang Q., Teng F., McLellan B.C. (2022). A Critical Review on Deployment Planning and Risk Analysis of Carbon Capture, Utilization, and Storage (CCUS) toward Carbon Neutrality. Renew. Sustain. Energy Rev..

[88] Moon S., Lee S., Ahn Y.H., Park Y. (2021). Abnormal Thermodynamic Promotion and Tuning Behavior of Epoxycyclopentane for Its Implications in CO_2_ Storage. Chem. Eng. J..

[89] Chakrabortty S., Kumar R., Nayak J., Jeon B.H., Dargar S.K., Tripathy S.K., Pal P., Ha G.S., Kim K.H., Jasiński M. (2023). Green Synthesis of MeOH Derivatives through in Situ Catalytic Transformations of Captured CO_2_ in a Membrane Integrated Photo-Microreactor System: A State-of-Art Review for Carbon Capture and Utilization. Renew. Sustain. Energy Rev..

[90] Das S., Wan Daud W.M.A. (2014). A Review on Advances in Photocatalysts towards CO_2_ Conversion. RSC Adv..

[91] Chen Z., Zhang G., Cao S., Chen G., Li C., Izquierdo R., Sun S. (2023). Advanced Semiconductor Catalyst Designs for the Photocatalytic Reduction of CO_2_. Mater. Rep. Energy.

[92] Xu Z., Zheng X., Ji B., Bao S., Mei J., Yang Z., Rong J., Li Z. (2025). Development of cerium-doped porous composite aerogel using cellulose nanocrystals for enhanced CO_2_ capture and conversion. J. Colloid Interface Sci..

[93] Montini T., Melchionna M., Monai M., Fornasiero P. (2016). Fundamentals and Catalytic Applications of CeO2-Based Materials. Chem. Rev..

[94] Kumar A., Singh P., Khan A.A.P., Van Le Q., Nguyen V.H., Thakur S., Raizada P. (2022). CO_2_ Photoreduction into Solar Fuels via Vacancy Engineered Bismuth-Based Photocatalysts: Selectivity and Mechanistic Insights. Chem. Eng. J..

[95] Lei W., Zhang T., Gu L., Liu P., Rodriguez J.A., Liu G., Liu M. (2015). Surface-Structure Sensitivity of CeO_2_ Nanocrystals in Photocatalysis and Enhancing the Reactivity with Nanogold. ACS Catal..

[96] Xu C., Qu X. (2014). Cerium Oxide Nanoparticle: A Remarkably Versatile Rare Earth Nanomaterial for Biological Applications. NPG Asia Mater..

[97] Masika E., Mokaya R. (2013). High Surface Area Metal Salt Templated Carbon Aerogels via a Simple Subcritical Drying Route: Preparation and CO_2_ Uptake Properties. RSC Adv..

[98] Jeon D.H., Min B.G., Oh J.G., Nah C., Park S.J. (2015). Influence of Nitrogen Moieties on CO2 Capture of Carbon Aerogel. Carbon Lett..

[99] He P., Qian X., Fei Z., Liu Q., Zhang Z., Chen X., Tang J., Cui M., Qiao X. (2018). Structure Manipulation of Carbon Aerogels by Managing Solution Concentration of Precursor and Its Application for CO_2_ Capture. Processes.

[100] Hu Y., Tong X., Zhuo H., Zhong L., Peng X., Wang S., Sun R. (2016). 3D Hierarchical Porous N-Doped Carbon Aerogel from Renewable Cellulose: An Attractive Carbon for High-Performance Supercapacitor Electrodes and CO_2_ Adsorption. RSC Adv..

[101] Wang C., Jiang W., Jiang G., Zhang T., He K., Mu L., Zhu J., Huang D., Qian H., Lu X. (2023). Machine Learning Prediction of the Yield and BET Area of Activated Carbon Quantitatively Relating to Biomass Compositions and Operating Conditions. Ind. Eng. Chem. Res..

[102] Chang J., Lee J.-Y. (2024). Machine Learning-Based Prediction of the Adsorption Characteristics of Biochar from Waste Wood by Chemical Activation. Materials.

[103] Vishnyakov A. (2025). Machine Learning in Computational Design and Optimization of Disordered Nanoporous Materials. Materials.

[104] Tafreshi O.A., Saadatnia Z., Ghaffari-Mosanenzadeh S., Okhovatian S., Park C.B. (2022). Machine learning-based model for predicting the material properties of nanostructured aerogels. SPE Polym..

[105] Jiang W., Xing X., Li S., Zhang X., Wang W. (2019). Synthesis, characterization and machine learning based performance prediction of straw activated carbon. J. Clean. Prod..

